# Attributable Fraction of Cancer Related to Occupational Exposure in Italy

**DOI:** 10.3390/cancers15082234

**Published:** 2023-04-10

**Authors:** Giulia Collatuzzo, Federica Turati, Matteo Malvezzi, Eva Negri, Carlo La Vecchia, Paolo Boffetta

**Affiliations:** 1 Department of Medical and Surgical Sciences, University of Bologna, 40126 Bologna, Italy; 2 Department of Clinical Sciences and Community Health (DISCCO), University of Milan, 20122 Milan, Italy; 3 Stony Brook Cancer Center, Stony Brook University, Stony Brook, NY 11794, USA

**Keywords:** cancer, occupation, occupational carcinogens, occupational exposure, attributable fraction, epidemiology

## Abstract

**Simple Summary:**

We estimated the number of cancersdue to exposure to occupational carcinogens in Italy. To calculate the attributable fraction of cancer, we used as counterfactual scenario the absence of exposure, and considered prevalence of exposure from 15–20 years prior than cancer to account for the latency. Large-scale studies and meta-analyses were the source of data on relative risk and exposure prevalence. We found that UV radiation, diesel exhaust and wood dust and silica dust were the most prevalent occupational carcinogens. About 60% of cancer in Italy were attributable to asbestos, and mesothelioma had the largest attributable fraction to occupational exposure. Overall, 0.9% of cancer cases and 1.6% of cancer deaths were attributable to occupational carcinogens in Italy, indicating the importance of maintaining a high level of surveillance of carcinogens at the workplace.

**Abstract:**

Background: Exposure to occupational carcinogens is an important and avoidable cause of cancer. We aimed to provide an evidence-based estimate of the burden of occupation-related cancers in Italy. Methods: The attributable fraction (AF) was calculated based on the counterfactual scenario of no occupational exposure to carcinogens. We included exposures classified as IARC group 1 and with reliable evidence of exposure in Italy. Relative risk estimates for selected cancers and prevalences of exposure were derived from large-scale studies. Except for mesothelioma, a 15–20-year latency period between exposure and cancer was considered. The data on cancer incidence in 2020 and mortality in 2017 in Italy were obtained from the Italian Association of Cancer Registries. Results: The most prevalent exposures were UV radiation (5.8%), diesel exhaust (4.3%), wood dust (2.3%) and silica dust (2.1%). Mesothelioma had the largest AF to occupational carcinogens (86.6%), followed by sinonasal cancer (11.8%) and lung cancer (3.8%). We estimated that 0.9% of cancer cases (N~3500) and 1.6% of cancer deaths (N~2800) were attributable to occupational carcinogens in Italy. Of these, about 60% were attributable to asbestos, 17.5% to diesel exhaust, followed by chromium and silica dust (7% and 5%). Conclusions: Our estimates provide up-to-date quantification of the low, but persistent, burden of occupational cancers in Italy.

## 1. Introduction

Occupation-related cancers are largely avoidable. In 2017, a total of 47 agents were identified as occupational carcinogens, belonging to group 1, by the International Agency for Research on Cancer (IARC) [1].

Since Pott’s evidence of scrotal skin cancer occurring in chimney sweeps, linked to the intense and prolonged exposure to soot, occupational epidemiology has provided evidence on the causal relationship between certain occupational carcinogens and different cancer types [2]. A well-known causal association is that between asbestos and mesothelioma. While epidemiology investigations collect evidence on additional possible carcinogens found in work settings, health policies and government regulations have been introduced, and occupational exposure limits have been set for several chemical agents. Exposure limits are heterogeneous among the different countries. Italy adopted stringent regulations, aimed at prevention of occupation-related cancer. For example, of the known occupational carcinogens, Italy has seen a progressive reduction in the use of aromatic amines, which in the past represented a serious hazard in certain industrial sectors, mainly the dye industry [3]. Regarding asbestos, its use was banned in 1992, but large proportions of workers—especially in construction and shipyard fields—had been exposed in previous years [4]. The consequences of past exposure to asbestos are a present medical issue, and the risk still exists for categories of workers such as renovators and asbestos disposal workers. 

Occupational exposures may also interact with other carcinogens, e.g., tobacco smoking and alcohol drinking, leading to a combination of effects based on the mechanisms of interaction of the two risk factors. 

In Italy, workers represent the 60% of the total population in 2022 [5].

As part of a systematic assessment of the causes of cancer in Italy, the present study aimed to provide an evidence-based estimate of the proportion of cancer incidence in 2020 and mortality in 2017 attributable to occupational carcinogens in Italy.

## 2. Methods

We calculated the attributable fraction (AF) using as alternative (counterfactual) scenario the absence of exposure. In most instances, the latency time between exposure and cancer is not known. We considered a latency period of about 15–20 years between exposures and cancer occurrence. Therefore, using data on cancer incidence in 2020 and on cancer mortality in 2017, the priority was given to exposure prevalence data from around 2000. 

With regard to incidence data, it is possible that the COVID-19 pandemic had, in part, skewed cancer incidence. This may have been due to (i) deaths of cancer patients from COVID-19 infection before cancer diagnosis or (ii) underdiagnosis linked to the reduction of cancer screening participation. These issues would have resulted in a reduction of the diagnosis of new cancer cases. We recommend readers to analyze the results with this awareness. 

We included occupational exposures classified as Group 1 agents by IARC [1]. We did not consider occupational carcinogens, like such as mustard gas and chloro-methyl ethers, which have not been used in recent decades (see Supplementary Table S1 for details). For this study, we considered all the agents for which an estimate of the prevalence of occupational exposure in 1990–2000 in Italy was available. We selected the following risk-outcome pairs: formaldehyde and nasopharyngeal cancer and leukemia; wood dust and sinonasal cancer; strong inorganic acid mists and laryngeal cancer; polycyclic aromatic hydrocarbons (PAH)and laryngeal, lung and bladder cancer; silica dust and lung cancer; asbestos and lung and ovarian cancer and mesothelioma; diesel exhaust and lung and bladder cancer; arsenic and lung cancer; chromium and lung cancer; cadmium and lung cancer; nickel and lung cancer; trichloroethylene (TCE) and kidney cancer; benzene and leukemia. We did not estimate the AF for perchloroethylene (PCE) and working as a painter, working in iron and steel foundries, or exposure to welding fumes and rubber because of lack of available data on their prevalence in the Italian working population. We also did not examine aromatic amines (associated with bladder cancer and lung cancer) and vinyl chloride (associated with liver angiosarcoma) because of the very uncommon use of these products in the 2000s in Italy.

The numbers of exposed workers were derived from the CAREX Italy study [6]. In this study, the number of workers exposed to occupational carcinogens was based on national labor force data by industry in 2001, coupled with reference exposure prevalence data from Finland and the United States. These preliminary estimates were reviewed and corrected by national experts. To obtain the prevalence of exposure, the number of exposed workers derived from CAREX [6] were divided by 36,036,000 (the number of residents aged 18–65 in Italy in 2001) and multiplied by 3, the average number of jobs held by controls in a large-scale case–control study of occupational factors for head and neck cancer [7]. The estimates of prevalence of exposures used in the current study are reported in Table 1. Since data on the prevalence of exposure were not stratified by sex, we applied them to the whole Italian adult population. For all occupational agents, exposure was dichotomous (ever/never) and the alternative (counterfactual) exposure scenario is that of never occupational exposure.

For all agents except mesothelioma from asbestos, we calculated the AF based on the method originally described by Levin [25] for dichotomous exposure variables:

$$\mathrm{AF}=\frac{P\times \left(\mathrm{RR}-1\right)}{\left[P\times \left(\mathrm{RR}-1\right)\right]+1}$$

which is based on the combination of estimates of relative risk (RR) and prevalence of exposure (P). For mesothelioma attributable to asbestos, we used the prevalence of cases with occupational exposure reported by the Italian National Mesothelioma Registry (ReNaM) [10]. We derived from the Registry two estimates, the primary one based on cases classified as certainly, probably and possibly exposed to asbestos at occupational level, and the secondary estimate which also included cases with an unknown source of exposure.

We extracted the RR of cancers for occupational carcinogens from recent meta-analyses or pooled analyses (Table 1). As expected, most of the RR derived from studies conducted on men. Where we did not found information on the proportion of females to males in key industries in Italy, we assumed the same RR in women, except for ovary cancer. 

We did not select latency to match the average length of follow-up in the RR considered, because as this information was not available in the sources of data used for RR (Table 1). 

We estimated the number of deaths and cases of cancers attributable to occupational agents in Italy by applying AF estimates to data on cancer mortality and incidences obtained from the 2020 Report of the Italian Association of Cancer Registries (AIRTUM) [26]. We developed formulae to estimate the number of deaths and cases of cancers not included in the AIRTUM Report, namely nasopharyngeal, sinonasal, laryngeal cancer and mesothelioma (details are given in the Appendix A Table A1). By using exposure estimates from 2001 and cancer data in 2017 (for mortality) and 2020 (for incidence), we accounted for a 15–20-year lag, which is considered relevant for most occupational carcinogens. One exception was mesothelioma, whose AF was estimated from the National Mesothelioma Registry [10], which takes into consideration the whole occupational and non-occupational exposure history of patients. 

## 3. Results

As shown in Table 1 [8,9,11,12,13,14,15,16,17,18,19,20,21,22,23,24], the most prevalent occupational carcinogens were UV radiation (5.8%), diesel exhaust (4.3%), wood dust (2.3%) and silica dust (2.1%); the other agents had a prevalence of exposure <2%. Consistently, AF% for sinonasal (11.8%) and lung cancer (3.8%) were among the highest ones, the first related to wood dust and the second to diesel exhaust and silica dust (Table 2). The highest AF% was observed for mesothelioma, i.e., 86.6%, when considering cases probably and possibly exposed to asbestos at an occupational level and cases with unknown sources of exposure according to the National Mesothelioma Registry, and 69.2% when only considering cases probably and possibly exposed to asbestos at an occupational level. 

Overall, 1.6% cancer deaths and 0.9% cancer cases were attributable to occupational carcinogens in 2017–2020 in Italy. 

Lung cancer and mesothelioma were the cancers with the highest number of attributable deaths (1152 and 1623, respectively) and attributable cases (1390 and 1720, respectively). The secondary analysis of mesothelioma resulted in 1374 deaths and 1720 cases. 

Asbestos was responsible for the highest number of cancer deaths (1741) and cases (1869), followed by diesel exhaust (489 and 621), chromium (191 and 240), silica dust (143 and 173), arsenic (94 and 113) and PAH (84 and 137); the other agents accounted for a minority of cancer deaths, and only UV radiation caused a substantial number of cases (155). The details are reported in Table 3.

Table 4 illustrates the AF calculated by cancer type and occupational carcinogens, and the corresponding number of deaths and cases. Lung cancer was linked to eight different occupational agents, with diesel exhaust explaining 1.4% of the burden. We found that diesel exhaust exposure and PAH exposure were each responsible for 0.2% of bladder cancer; UV radiation was responsible for 1% of melanoma; wood dust, chromium and nickel accounted for 4.7%, 18.3% and 1.4% of sinonasal cancer, respectively; 0.6% leukemias were referrable to benzene and 0.4% to formaldehyde; 0.1% of laryngeal cancers to strong inorganic acid mists and 0.3% to PAH; and 0.3% of nasopharyngeal cancers to formaldehyde. Asbestos accounted for the large majority of mesotheliomas, and for 0.5% of the ovary cancers. 

The figures for Italy were markedly lower than those estimated for other countries, as shown in Table 5 [27,28,29,30,31,32,33,34,35,36,37,38,39]. 

## 4. Discussion 

This work offers a novel comprehensive estimate of the burden of workplace-related cancer in Italy. According to our results, work-related cancers account for 1.6% of cancer deaths and about 1% of cancer cases. Exposure to occupational agents as a cause of a large proportion of respiratory cancers is widely recognized [1,40,41]. These figures are markedly smaller than those related to other risk factors, such as tobacco smoking [42] and infections [43]. 

We included most of the main previous occupational carcinogens in Italy. We could not give estimates for welding fumes, painting, iron and steel foundries, and the rubber industry, because of lack of representative prevalence data in the Italian population. For all cancer types except mesothelioma, the estimates we presented were based on exposures derived from the job titles rather than from workplace measurements, do not account for dose–response relationship, and relate to exposures that occurred 20 years prior to the incidence and mortality data; the data reported by the 2001 Censuses of Industry, Services and Agriculture (CAREX) account for the latency time between exposure and cancer diagnosis [6]. We provided two estimates of AF for mesothelioma due to occupational exposure to asbestos, based on different criteria of exposure classification by the ReNaM [10]. They should be interpreted as the boundaries of the range of plausible values of the burden of mesothelioma due to occupation. Incidence and mortality may have distinct latency times from carcinogen agent exposures, and they can vary for certain carcinogen–cancer associations, thus representing a limitation in our study. 

In the past, exposure to diesel exhaust has been neglected, but has recently become the focus of several studies, with increasing evidence of its carcinogenicity for different organs. Primary sources of exposure include vehicles, ships, trains, oil and gas production facilities, shipyards, chemical manufacturing and electric utilities [44]. Given the heterogeneous origin of diesel exhaust exposure, and that several cofactors influence diesel exhaust concentration in the air (e.g., type of diesel fuel used, weather, conditions and age of the vehicle, space ventilation, use of protective personal equipment (PPE) and so on [26]), it is difficult to accurately assess the prevalence of diesel exhaust exposure. Currently, a threshold level for diesel exhaust carcinogenicity has not been identified, despite regulatory limits having been recently purposed [45]. Cancer risk related to diesel exhaust is expected to decrease, as new technology diesel engines are taking the place of old technology diesel engines [46]. 

Sinonasal cancer is an example of rare neoplasm which could be largely prevented through occupational surveillance. The use of PPE (e.g., masks) and presence of local exhaust ventilation have been demonstrated to be effective in the reduction of exposure to wood dust; however, some excess risk of nasal cancer still persisted in a previous Italian study [47]. Woodworking industries started providing their employees with PPE or installing exhaust systems only in 1981 [47]. Wood dust can be of two types: softwood and hardwood, the latter being the one responsible for sinonasal cancer. Few studies consider such a distinction [15]. This may account, at least in part, for the marked heterogeneity in risk estimates across different studies [48]. The IARC provides an evaluation of carcinogenicity for wood dust as a whole (both soft and hard), while the European Union (EU) has classified only hardwood dust as a carcinogen (directive no. 1999/38/EC) [49]. Demers et al. estimated a RR of 3 [15], considering the presence of both softwood and hardwood dust in workplaces. When looking at hardwood dust singularly, the RRs are much higher than for softwood [15]. It was not possible to distinguish between workers exposed to softwood (e.g., workers employed in sawmills in northern Italy) and those exposed to hardwood dust (e.g., furniture makers) in this study. Workers exposed in Italy to wood dust (both soft and hard) were first estimated by the WoodEx project at about 351,000 in the period 2000–2003 [50]. Subsequently, on the basis of the Italian Information System on Occupational Exposure to Carcinogens (SIREP) from 31 December 2011, and only for some specific sectors, the number of workers potentially exposed to hardwood dust in Italy were estimated to be around 117,000. 

AF estimates are dynamic because of the change in the prevalence of the exposure to various agents over time, and of new emerging evidence on the causal role of a suspected human carcinogen for a certain cancer type, that may lead to the need to account for additional risk factors not considered in AF estimates. In addition, the carcinogens we included in the present analysis may be related to other cancer types, although the current evidence is considered insufficient (e.g., diesel exhaust and kidney cancer) [20,51,52]. Thus, the current results may underestimate the burden of cancer attributable to occupational risk factors. 

It is debatable whether suspected occupational carcinogens, such as those listed in Group 2A of the IARC Monograph evaluations, should be included in an estimate of attributable cancers. Consistently with previous estimates [27,34,53], we calculate the AF only for those occupational risk factors defined as Group 1 human carcinogens according to the IARC. Rushton et al. [28] presented results on cancer deaths which were increased by about one third after including suspected carcinogens. A more recent study by Brown et al. [29] found 5% of all cancer in UK to be attributable to occupation; however, the study considered job titles rather than specific occupational exposures, therefore its results ae not directly comparable to ours. We have added the cases attributed to different agents and ignored the fact that the same workers may have been exposed to several carcinogens, while the ideal approach would consider the combination of concomitant exposures to different occupational carcinogens and their interactions [54]. Nevertheless, data on the prevalence of combinations of occupational risk factors are lacking, and very difficult to collect [54]. Lung cancer is one of the most frequent and deadly cancers globally and in Italy, and besides tobacco smoking, can be caused by multiple factors [55]. Often, these factors co-occur in the same population, as in the case of many workers [56,57,58,59]. The analysis performed to calculate the AF of lung cancer was particularly complex due to the multiple agents involved. To better address this issue, we considered the proportion of the different factors as acting independently. Independence assumes a multiplicative model. This may lead to an overestimation of RR and consequently attributable risks since some exposures may involve similar carcinogens and hence lead to some multiplicative or additive RR. However, the available data on exposure do not allow us to separate additive interactions, which can be considered by using adequately adjusted RR, and synergistic interactions, which are harder to quantify from a single RR [60,61,62].

Some of the RR estimates used to calculate the AF derive from old studies, despite prioritizing more recent RR values. Such older estimates may no longer be relevant to exposure circumstances determining the current burden of cancer [63], and may have led to an overestimation of the AF. Few studies accounted for the intensity of exposure when calculating the RR between risk factors and cancer [64], which may have either increased or reduced the burden of cancer we calculated. Another factor which may have impaired the accuracy of our estimates is that most of the RR were not adjusted for smoking and other confounding factors [54]. This could have especially overestimated the AF of smoke-related cancers, such as lung, laryngeal and bladder cancers. An additional limitation is that the RR mainly referred to men, except that for asbestos and ovarian cancer. Women and men belonging to the same working group may cover different roles, with “typically male” working activities being more exposed to occupational carcinogens than those “not typically male”. This may have led to an overall overestimate of the AF of cancers. Moreover, the RR were derived from studies conducted in other countries than Italy. 

While a previous study on the AF of occupation-related cancer in France [33] did not include UV radiation among the risk factors, this analysis provided data on this important carcinogen. According to our results, and consistently with data on the relatively low fatality of malignant melanoma, UV radiation had more impact on the estimate of attributable cases than that of deaths. The AF of non-melanoma skin cancers related to occupational UV radiation was 6.3% in 2011 in Canada [65]. A recent study calculated that 27.4% of the attributable burden of disease due to occupational exposure to UV radiation was represented by melanoma [66]. Residual confounding effects may derive from recreational exposure to the sun, and by the adoption of sun protection behaviors. In addition, different predispositions to melanoma and non-melanoma skin cancer, like family history and skin complexion, play a role [67]. While different factors may have influenced our results, it is likely that the figures shown represent the minimum fraction of melanomas attributable to UV radiation in workers, as this kind of exposure is often underestimated [68]. 

Overall, we estimated a lower proportion of cancers attributable to occupational exposure than those reported in other studies. A possible explanation is the different methodology adopted, in particular the use of dated sources for the RR by other studies [55], which reflected the past situation, connoted by heavier exposures. This may explain the lower AF we obtained in comparison to previous studies. 

All in all, we believe that our estimates show a reasonably accurate picture of occupational cancers in Italy, balancing the potential limitations which may have skewed the results (e.g., underdiagnosis of cancer in 2020 due to COVID-19 pandemic, and overweighed cancers in women). 

The exposure data from the UK study were mainly based on the CAREX database [6], which is also the reference we used for the present analysis. The lower prevalence of exposure to occupational carcinogens has already been pointed out by Mirabelli et al. when publishing the CAREX estimates [6], conveying the message of the need to overcome the traditional assumption of occupational carcinogens as the cause of substantial proportions of cancer deaths and cases, due to the progressive improvement in limiting hazardous exposures in the workplace, which was indeed successful in Italy. 

Aromatic amines were, in fact, not accounted for in our analysis, given the very low exposure registered in Italy over recent decades [69,70]. Next to the dramatic reduction in the use of aromatic amines in Italian industries starting around 1955 [69,70], the banning of asbestos in the early 1990s should be noted. The high proportions of mesothelioma cases and deaths calculated for the year 2020 were not unexpected; our results are, indeed, consistent with the prediction by Oddone and coworkers, who expected 1122 cases of mesothelioma attributed to asbestos in 2021 in Italy, despite not focusing on occupational exposure [71]. Indeed, the risk of mesothelioma persists throughout the lifespan of exposed subjects [72], and, while the banning prevented from the further use of asbestos, many asbestos residuals still need to be replaced from workplaces [73]. On the one hand, it is possible that our estimates undervalue the true number of cases and deaths from mesothelioma and ovarian cancers, given the particularly long latency of the development of the disease and the higher prevalence of asbestos which Italian workers were exposed to in the decades before our prevalence estimates. On the other hand, based on different publications, this is unlikely to be the case: the estimates provided by Scarselli et al. [74] reported that after asbestos ban (1996–2016) the exposure was almost halved compared with that reported in the CAREX study [6]. Italy was not included in the last Global Burden of Disease (GBD) analysis on mesothelioma due to occupational exposure to asbestos [75]. An additional difficulty when assessing the AF of mesotheliomas from occupational exposure to asbestos is its long latency [76], which would fall in a period when the prevalence of exposure was not yet measured in Italy. Thus, an accurate estimate of AF of mesothelioma in Italian workers was that based from the ReNaM [10], which classifies the mesotheliomas according to an individual-based risk assessment. Anyway, ReNaM recorded a substantial portion of mesotheliomas as undefined, because of the lack of sufficient evidence of a causal relationship with environmental or workplace asbestos exposure. This could underestimate the actual prevalence of occupational asbestos exposure in Italy, and consequently the AF of the related cancers. Another reason why the proportion of mesothelioma classified as occupation-related by the ReNaM [10] is relatively low is that a role is also played by environmental exposure to asbestos [77,78,79]. According to our primary estimate of asbestos-related mesotheliomas, including cases of undefined origin, the AF was 86.6%. It is likely that the AF of mesotheliomas related to occupational exposure to asbestos ranges among the figures we estimated. 

Given the need for further evidence on the causes of work-related cancers [1], and as most workplace exposures have not been evaluated for their carcinogenic potential due to the paucity of quantitative exposure data [1], the precise proportions of cancer due to occupation-related exposure remains in part undefined. 

Besides these hypotheses, our results suggest the successful control of occupational carcinogens in Italy, resulting in low rates of cancer due to work exposures [80]. 

Several health policies have been introduced in Italy since the 1970s, and the profile of occupational medicine and occupation-related cancers has changed in the subsequent decades [80]. 

When estimating the AF of occupation-related cancers, in an ideal setting, the route of exposure should be accounted for [81]. The dominant routes of exposure are inhalation and dermal contact [1]. Different effects could be exerted by the same substance in different organs, and also depending on the type of contacts which the worker has with that substance [82]. An example is that of asbestos, which causes mesothelioma based on the dimension of the fibers when inhaled, but does not constitute a carcinogenic agent in the case of cutaneous contact or ingestion [83]. Similarly, it is possible that some known substances may exert a carcinogen effect through a route of exposure which has not yet been investigated, or not investigated enough to provide sufficient evidence of its carcinogen potential [84]. 

Despite the limitations of the present analysis, and despite the estimates of work-related cancers being mostly conservative since they are based on likely underestimated prevalences of carcinogens exposures in the workplaces, our results indicate that occupation-related cancers currently represent a small proportion of cancers in Italy; this is mainly due to the progressive reduction in the prevalence of exposure over time, as previously demonstrated by Mirabelli et al. [6]. Our death estimates are also far smaller than those calculated in a previous study for the year 2006, which were estimated to be around 8000–8500 [84]. Another previous estimate found broader estimates of occupational/related cancers in Italy [85]. Another possible reason for the smaller number observed in this analysis is the redistribution of cancer occurrence, reflected also in the number of cancer deaths, in the Italian population [80]. For example, cancer types other than those we included in the present analysis, namely the non-occupational ones, may represent a larger proportion of the total cancers in Italy than elsewhere, leading to a lower AF of occupation-related cancers [86]. 

## 5. Conclusions

Occupational cancers are largely preventable, and the figures we presented depict the successful efforts which Italy has been making in controlling the health of its working population. The reduced figures of the AF of occupation-related cancers in Italy are mainly attributable to the progressive reduction in the prevalence of exposure to the main occupational carcinogens. The decreasing proportion of workers exposed to asbestos in recent years is likely to further reduce these numbers in the near future [71]. The reinforcement of occupational health recommendations, including the promotion of PPE use and the education about health hazards and their prevention at the workplace, are a key point to lower the number of workers exposed to occupational carcinogens such as wood dust, nickel, and UV radiation, whose control still needs improvement. 

Moreover, exposure thresholds for diesel exhaust emissions should be introduced to further prevent lung and bladder cancers, and possibly other cancers too. 

Older generations of workers may still be at higher risk of developing work-related cancers, due to exposures occurring in earlier periods [70,86]. 

Our data have important legal implications, given the possibility for occupation-related diseases to be compensated for based on the Italian Workers’ Compensation Authority (INAIL) [87].

## Figures and Tables

**Table 1:** Prevalence of exposure of the occupational agent included in the analysis and relative risk (RR) for cancers associated with them.

Agent	Prevalence of Exposure (%) *	Cancer	RR (95% CI)	Reference for RR
Silica dust, crystalline	2.1	Lung	1.20 (1.12–1.28)	Steenland 2001 [8]
Asbestos	0.87 ^¶^	Lung	1.48 (1.44–1.52)	Goodman 1999 [9]
		Mesothelioma	NA	ReNaM 2022 [10]
		Ovary	1.77 (1.37–1.28)	Camargo 2011 [11]
Strong inorganic acid mist	0.45	Larynx	1.21 (0.87–1.67)	IARC 2012 ^†^ vol 100F [12]
PAH	1.01	Larynx	1.30 (1.07–1.58)	Rota et al., 2014 ** [13]
		Lung	1.20 (1.02–1.41)	Rota et al., 2014 ** [13]
		Bladder	1.18 (1.01–1.37)	Rota et al., 2014 ** [13]
Diesel exhaust	4.3	Lung	1.33 (1.21–1.46)	Lippsett 1999 [14]
		Bladder	1.04 (1.01–1.07)	Meta-analysis of cohort studies on occupational diesel exhaust exposure conducted by Collatuzzo et al.
Wood dust	2.33	Sinonasal	3.1 (1.6–5.6)	Demers 1995 [15]
Benzene	1.53	Leukemia	1.40 (1.23–1.57)	Khalade 2010 [16]
Formaldehyde	0.94	Nasopharynx	1.33 (0.69–2.56)	Bosetti 2008 [17]
		Leukemia	1.39 (1.15–1.68)	Bosetti 2008 [17]
TCE	0.28	Kidney	1.32 (1.17–1.50)	Karami 2012 [18]
Arsenic	0.27	Lung	2.04 (1.90–2.19)	Hayes 1997 ** [19]
Chromium	1.30	Sinonasal	8.0 (4.3–13.6)	IARC 2012 ^†^ vol 100C [20]
		Lung	1.41 (1.35–1.47)	Cole 2005 [21]
Cadmium	0.40	Lung	1.42 (0.91–2.23)	Chen 2016 [22]
Nickel	0.81	Sinonasal	2.8 (1.2–5.5)	Hayes 1997 ** [19]
		Lung	1.12 (1.05–1.20)	Behrens 2018 [23]
UV radiation	5.84	Melanoma	1.18 (1.01–1.38)	Togawa 2021 ^‡^ [24]

Notes: PAH = polycyclic aromatic hydrocarbons; TCE = tetrachloroethylene. * Source: CAREX paper. ^¶^ Direct estimate of the attributable fraction (AF) from the ReNaM (AF = 0.866). ^†^ Meta-analysis of studies included in IARC Monographs. ^‡^ Results for farmers ** Meta-analysis of studies included in the reference.

**Table 2:** Attributable fraction (AF), and number of deaths in 2017 and cases in 2020 attributable to occupational carcinogens in Italy by cancer type.

Cancer	AF%	Attributable Deaths	Attributable Cases
Nasopharynx (C11)	0.3	1	2
Sinonasal (C30–31)	11.8	17	34
Larynx (C32)	0.4	6	13
Lung (C33–34)	3.8	1152	1390
Mesothelioma (C45) *	86.6	1623	1720
Melanoma (C43)	1.0	22	155
Ovary (C56)	0.5	16	25
Bladder (C67)	0.4	23	92
Kidney (C64–C66, C68)	0.09	4	12
Leukemia (C91–C95)	0.8	48	61
Total	-	2912	3594
% of total cancer	-	1.6	0.9

* Notes: according to the alternative estimates for mesothelioma (AF% = 69.2), the number of attributable deaths was 1623 and that of attributable cases was 1720.

**Table 3:** Number of deaths in 2017 and cases in 2020 attributable to occupational carcinogens in Italy by agent.

Occupational Carcinogen	No. Deaths	No. Cases
Silica dust, crystalline	143	173
Asbestos *	1741	1869
Strong inorganic acid mists	1	3
PAH	84	137
Diesel exhaust	489	621
Wood dust	7	14
Benzene	38	49
Formaldehyde	24	31
TCE	3	12
Arsenic	94	113
Chromium [VI]	191	240
Cadmium	53	64
Nickel	35	44
UV radiation	21	155

Notes: PAH = polycyclic aromatic hydrocarbons; TCE = tetrachloroethylene. * According to the alternative estimates for mesothelioma (AF% = 69.9), the number of deaths attributable to asbestos was 1416 and that of cases attributable to asbestos was 1523.

**Table 4:** Population attributable fractions (AF) by cancer type and occupational carcinogen in Italy. N = number.

Occupational Carcinogens and Related Cancers	AF%	No. Attributable Deaths in 2017	No. Attributable Cases in 2020
***Nasopharynx***			
Formaldehyde	0.3	0.8	1.6
***Sinonasal***			
Wood dust	4.7	6.7	13.6
Chromium	8.3	11.9	24.3
Nickel	1.4	2.0	4.2
***Larynx***			
Strong inorganic acid mists	0.1	1.5	3.1
PAH	0.3	4.9	10.0
***Lung***			
Silica dust	0.4	143.3	172.8
Asbestos	0.3	102.7	123.8
PAH	0.2	68.7	82.9
Diesel exhaust	1.4	478.4	576.9
Arsenic	0.3	93.7	188.6
Chromium	0.5	179.6	216.5
Cadmium	0.08	26.7	32.1
Nickel	0.6	217.6	262.4
***Mesothelioma***			
Asbestos	86.6 ^¤^	1623.0 ^¤^	1719.9 ^¤^
	69.2 ^§^	1296.9 ^§^	1374.3 ^§^
***Melanoma***			
UV radiation	1.0	21.5	154.7
***Ovary***			
Asbestos	0.5	16.2	25.1
***Bladder***			
PAH	0.2	10.8	43.9
Diesel exhaust	0.2	10.8	44.2
***Kidney***			
TCE	0.09	3.4	12.2
***Leukemia***			
Benzene	0.6	38.1	48.5
Formaldehyde	0.4	22.9	29.1

Notes: PAH = polycyclic aromatic hydrocarbons; TCE = tetrachloroethylene. ^¤^ Based on the National Mesothelioma Registry including cases with unknown source of exposure. ^§^ Based on cases classified by the National Mesothelioma Registry as certainly, probably and possibly related to occupational asbestos exposure.

**Table 5:** Attributable fraction of overall, lung and bladder cancers related to occupational carcinogens according to selected literature.

						Attributable Fraction		
Reference	Population	Method	Indicator	Time Period		All Cancers	Lung	Bladder
			Estimates based on relative risks and data on prevalence of exposure					
[27]	Nordic countries	Relative risks from review of literature, prevalence of exposure from national surveys	Incidence	~2000		3%	13%	2%
[28]	United Kingdom	Relative risk from meta- and pooled analyses, exposure prevalence mainly from national surveys	Mortality	2004		6.0%	16.5%	1.3%
[29]	United Kingdom	Relative risk from meta- and pooled analyses, exposure prevalence mainly from national surveys	Incidence	2015		5.0%	20.5%	7.1%
[30]	Brazil	Relative risk from meta- and pooled analyses, exposure prevalence mainly from national surveys	Incidence, Mortality	2020		2.3% in men, 0.3% in women	NA	NA
[31]	China	Relative risk and exposure prevalence from large-scale studies	Incidence, Mortality	2005		2.8% men 1.6% in women 3.1% in men, 2.1% in women	10.6% in men, 7% in women	10.6% in men, 11.4% in women
[32]	Western Europe	Average relative risks for eight carcinogens, prevalence of exposure from international data	Mortality	2000		NA	10%	NA
[33]	France	Relative risks from meta- and pooled analyses, exposure prevalence mainly from national surveys	Mortality	2000		4.0%	12.5%	5.5%
This study	Italy	Relative risk from meta- and pooled analyses, exposure prevalence mainly from national surveys	Incidence, Mortality	20172020		1.0%1.6%	4.2%	1.4%
			Estimates based on qualitative review of the literature					
[34]	United States	Critical review of literature	Mortality			6.8%	15%	10%
[35]	Various populations	Review of individual studies	Incidence, Mortality			NA	1–40%	0–24%
[36]	Finland	Included suspected carcinogens and all positive results	Incidence, Mortality	1996		13.8%	29.0%	14.2%
[37]	France	Attributable fraction from literature	Incidence, Mortality	1999		NA	13–29%	10–21.5%
[38]	United States	Attributable fraction from literature	Mortality	1997		NA	6.1–17.3%	7–19%
[39]	United Kingdom	Critical review of literature	Mortality			2% *	NA	NA

NA, not available. * men and women combined.

## Data Availability

The data that support the findings of this study are openly available from the authors upon reasonable request.

## Supplementary Materials

The following supporting information can be downloaded at: https://www.mdpi.com/article/10.3390/cancers15082234/s1. Table S1. Agents classified as established carcinogens excluded from the analysis.

## Appendix A

**Table A1:** Estimate of the number of cases of cancers for which no national data are available.

Cancer	Results	Justification, Reference
Nasopharyngeal cancer	8.7% (M), 6.9% (F) of oral and pharyngeal cancers	Based on distribution of incidence of subtypes of oral and pharyngeal cancer in 5 Italian cancer registries * [88]
Sinonasal cancer	8.8% (M), 33.3% (F) of laryngeal cancer	Based on ratio between sinonasal and laryngeal cancer in 5 Italian cancer registries * [88]
Laryngeal cancer	33.5% of UADT cancer incidence	Mortality available from AIRTUM [89]. Incidence based on distribution of mortality from UADT cancer [88]
Mesothelioma	5.3% of lung cancer mortality	Incidence available from AIRTUM [89]; mortality based on ratio between incidence of lung cancer and mesothelioma

* Milan; Veneto Region; Modena; Naples; Eastern Sicily. UADT: Upper aerodigestive tract.
