# Supplementary material for: Attributable Fraction of Cancer Related to Occupational Exposure in Italy
Source: Cancers (Basel). 2023 Apr 10;15(8):2234. doi: 10.3390/cancers15082234 (PMC10136839; doi:10.3390/cancers15082234)
Supplement: Supplementary file 1 [file cancers-15-02234-s001.zip › cancers-2181436-supplementary.pdf]

Supplementary Table S1. Agents classified as established carcinogens excluded from the analysis

**Agents or mixtures**	**Reason for exclusion**
Aromatic amines	No exposures in recent decades
Painters	No estimate of proportion of exposed workers
Welding fumes	No estimate of proportion of exposed workers
Talc containing asbestiform fibres	No estimate of proportion of exposed workers
Iron and steel foundry	No estimate of proportion of exposed workers
Rubber	No estimate of proportion of exposed workers
Beryllium	No exposures in recent decades
Coal tars and pitches	Included in polycyclic aromatic hydrocarbons
Shale oils	Included in polycyclic aromatic hydrocarbons
Soots	Included in polycyclic aromatic hydrocarbons
Vinyl chloride	No exposure in recent decades
Bis(chloromethyl)ether and chloromethyl methyl ether	No exposure in recent decades
4-Aminobiphenyl	Included in aromatic amines
Benzidine	Included in aromatic amines
2-Napththylamine	Included in aromatic amines
Ethylene oxide	Evidence for an association in humans is less than sufficient
Mustard gas	No exposure in recent decades
**Exposure circumstances**	
Aluminium production	Included in polycyclic aromatic hydrocarbons
Auramine manufacture	Included in aromatic amines
Boot and shoe manufacture and repair	Included in leather dust
Coal gasification	Included in polycyclic aromatic hydrocarbons
Coke production	Included in polycyclic aromatic hydrocarbons
Furniture and cabinet making	Included in wood dust
Hematite mining, underground, with radon exposure	Included in radon
Iron and steel founding	Included in polycyclic aromatic hydrocarbons and silica
Isopropanol manufacture	No exposure in recent decades
Magenta manufacture	Included in aromatic amines
